# Supplementary material for: Metabolic interplay between Proteus mirabilis and Enterococcus faecalis facilitates polymicrobial biofilm formation and invasive disease
Source: bioRxiv. 2024 Jun 4:2023.03.17.533237. Originally published 2023 Mar 18. Preprint. [Version 3] doi: 10.1101/2023.03.17.533237 (PMC10055233; doi:10.1101/2023.03.17.533237)
Supplement: Supplement 1 — Supplemental Figure 1. Proteomics identification of proteins present in P. mirabilis or E. faecalis single species biofilms. Protein identification was performed by searching against a combined database of P. mirabilis and E. faecalis protein sequence. Total protein intensities for single and polymicrobial biofilms. Protein intensities show that protein content within polymicrobial biofilms is largely driven by increases in P. mirabilis derived proteins. Supplemental Figure 2. Fitness of P. mirabilis argF, P. mirabilis speF, and E. faecalis arcD during growth in human urine. P. mirabilis and the argF mutant were co-cultured with either E. faecalis or the arcD mutant in human urine, and samples were plated every hour for determination of CFUs. A) P. mirabilis and B) E. faecalis CFU counts from the urine co-cultures. Error bars represent mean and standard deviation. *P<0.05, **P<0.01 by two-way ANOVA comparison of argF CFUs from argF+Ef to argF and wild-type CFUs from the other co-cultures in panel A, and for arcD CFUs compaed to E. faecalis CFUs in panel B. Supplemental Figure 3. Differences in biofilm biomass are not due to changes in bacterial viability. CFUs of biofilms grown for 24-hours in A) TSB-G or B) pooled human urine. Data represent the mean ± standard deviation for at least three independent experiments with at least two replicates each. ns = non-significant, * = P<.05 as determined by One-way ANOVA. Supplemental Figure 4. Colony forming units of each species from coinfected mice. P. mirabilis and E. faecalis bacterial counts in urine (U), bladder (B), kidney (K), and spleen (S) homogenates. The CFUs from an individual coinfected mouse are connected with a black line for each organ. [file media-1.pdf]

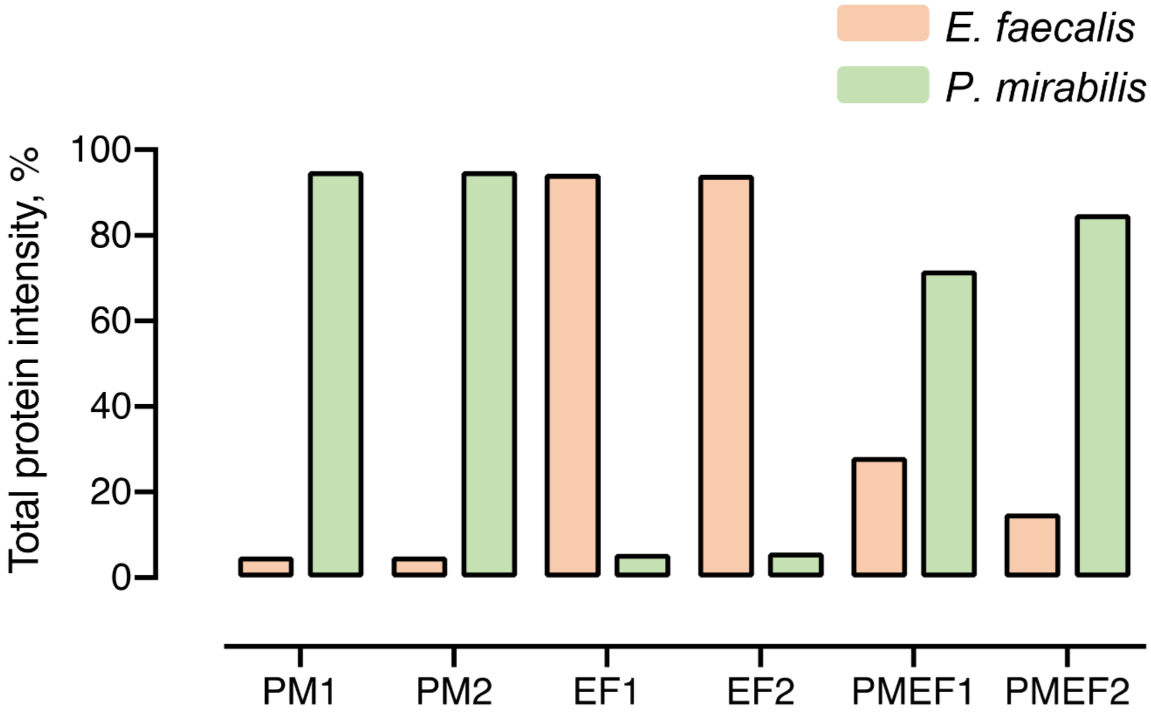

Supplemental Figure 1. Total protein intensities for single and *P. mirabilis* and *E. faecalis* polymicrobial biofilms. Protein identification was performed by searching against a combined database of *P. mirabilis* and *E. faecalis* protein sequence.

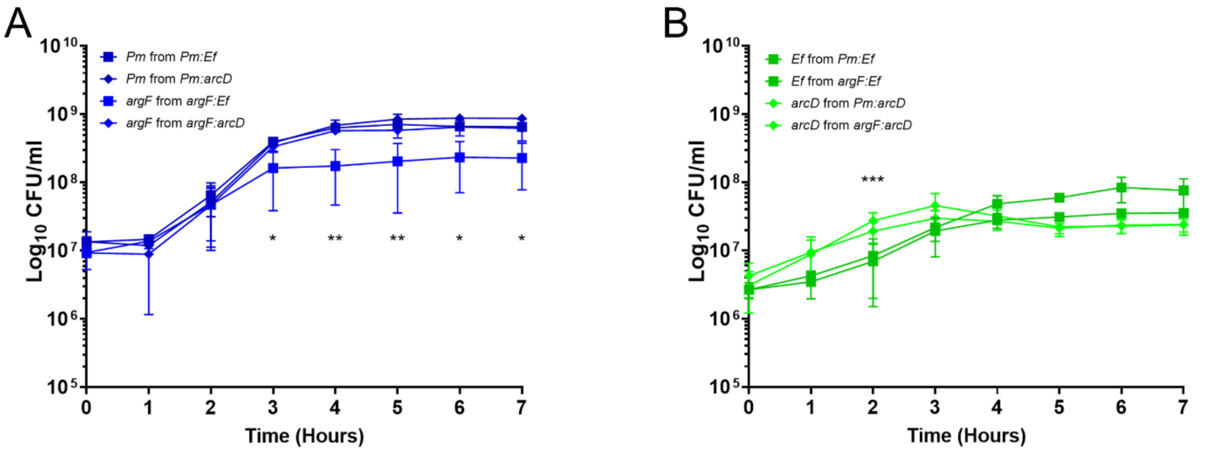

Supplemental Figure 2. Fitness of *P. mirabilis argF, P. mirabilis speF,* and *E. faecalis arcD* during growth in human urine. *P. mirabilis* and the *argF* mutant were co-cultured with either *E. faecalis* or the *arcD* mutant in human urine, and samples were plated every hour for determination of CFUs. A) *P. mirabilis* and B) *E. faecalis* CFU counts from the urine co-cultures. Error bars represent mean and standard deviation. *P<0.05, **P<0.01 by two-way ANOVA comparison of *argF* CFUs from *argF+Ef* to *argF* and wild-type CFUs from the other co-cultures in panel A, and for *arcD* CFUs compaed to *E. faecalis* CFUs in panel B.

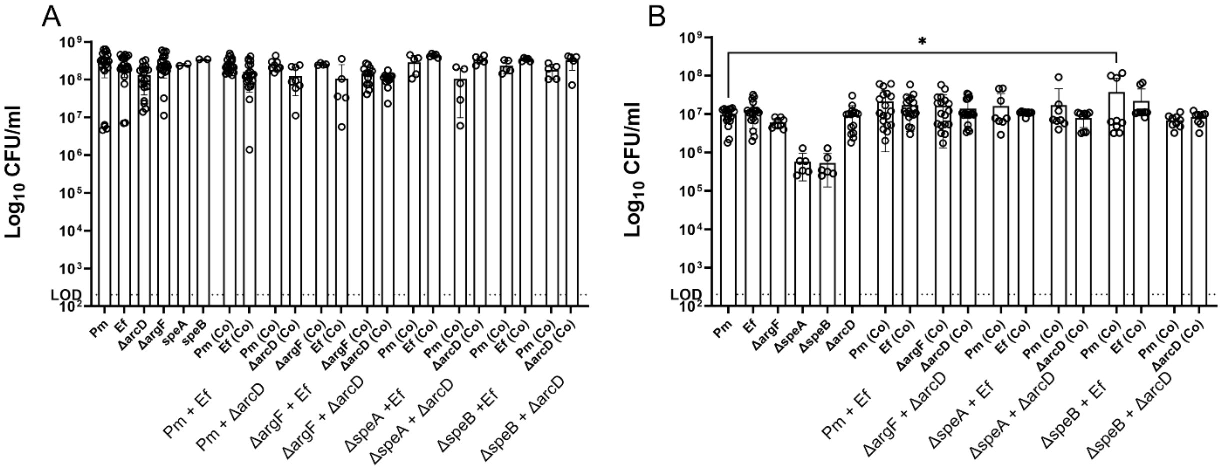

Supplemental Figure 3. Changes in colony forming units between wild-type and mutant biofilms is insignificant and not a driving factor for the differences in biofilm biomass. CFUs of biofilms grown for 24-hours in A) TSB-G or B) pooled human urine. Data represent the mean ± standard deviation for at least three independent experiments with at least two replicates each. ns = non-significant, * = P<.05 as determined by One-way ANOVA.

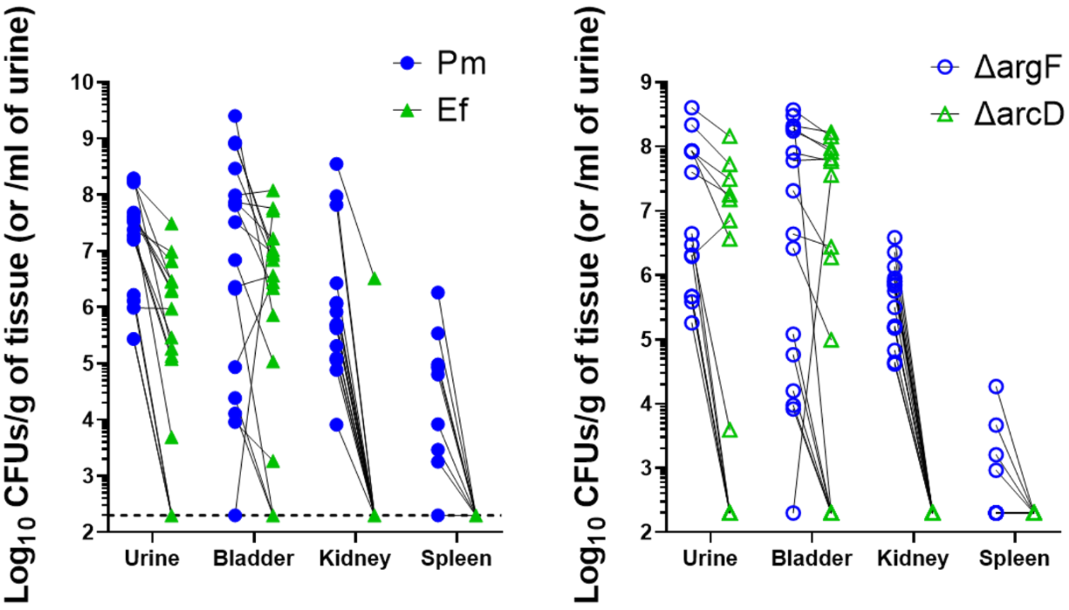

Supplemental Figure 4. Colony forming units of each species from coinfected mice. *P. mirabilis* and *E. faecalis* bacterial counts in urine (U), bladder (B), kidney (K), and spleen (S) homogenates. The CFUs from an individual coinfected mouse are connected with a black line for each organ.

Supplemental Item 5. LCMS methodology and analysis. *Protein digestion:* Biofilm suspension fraction (BS) was prepared as described above. After which, a surfactant-aided precipitation/on-pellet digestion method was adopted in the current study for sample preparation [82]. In brief, 100 µg protein was aliquoted from each sample and diluted to 1 µg/µL with 1% SDS. Protein was sequentially reduced by 10 mM dithiothreitol (DTT) at 56°C for 30 min and alkylated by 25 mM iodoacetamide (IAM) at 37°C in darkness for 30 min. Both steps were performed with rigorous vortexing in a thermomixer (Eppendorf). A total of 6 volumes of chilled acetone was then added to each sample with constant vortexing, and the mixture was incubated at -20°C for 3 hr. After centrifugation at 20,000 g, 4°C for 30 min, liquid was decanted, and protein pellet was gently washed by 500 µL methanol and air-dried for 1 min. A volume of 80 µL 50 mM pH 8.4 Tris-formic acid (FA) was then added, and samples were sonicated to loosen the protein pellet. A total volume of 20 µL trypsin (Sigma Aldrich, dissolved in 50 mM pH 8.4 Tris-FA) was added for 6-hr digestion at 37°C with rigorous vortexing in a thermomixer. Digestion was terminated by addition of 1 µL FA, and samples were centrifuged at 20,000 g, 4°C for 30 min. Supernatant was carefully transferred to LC vials for analysis.

LC-MS analysis: The LC-MS system consists of a Dionex µLtimate 3000 nano LC system, a Dinex µLtimate 3000 micro LC system with a WPS-3000 autosampler, and a ThermoFisher Orbitrap Fusion Lumos mass spectrometer. A large-inner diameter (i.d.) trapping column (300-um i.d. x 5 mm) was coupled to the nano LC column (75-um i.d. x 65 cm, packed with 2.5-um Xselect CSH C18 material) for high-capacity sample loading, cleanup and delivery. For each sample, 4 µL derived peptide was injected for LC-MS analysis. Mobile phase A and B were 0.1% FA in 2% acetonitrile (ACN) and 0.1% FA in 88% ACN. The 180-min LC gradient profile was: 4% for 3 min, 4–11 for 5 min, 11–32% B for 117 min, 32–50% B for 10 min, 50–97% B for 5 min, 97% B

for 7 min, and then equilibrated to 4% for 27 min. The mass spectrometer was operated under data-dependent acquisition (DDA) mode with a maximal duty cycle of 3 s. MS1 spectra was acquired by Orbitrap (OT) under 120k resolution for ions within the m/z range of 400-1,500. Automatic Gain Control (AGC) and maximal injection time was set at 120% and 50 ms, and dynamic exclusion was set at 45 s, ± 10 ppm. Precursor ions were isolated by quadrupole using a m/z window of 1.2 Th, and were fragmented by high-energy collision dissociation (HCD). MS2 spectra was acquired OT under 15k resolution with a maximal injection time of 50 ms. Detailed LC-MS settings and relevant information are enclosed in a previous publication by Shen et al.[83].

Data processing: LC-MS files were searched against a NCBI protein sequence database containing both *Proteus mirabilis* and *Enterococcus faecalis* protein sequences using Sequest HT embedded in Proteome Discoverer 1.4 (ThermoFisher Scientific). Target-decoy searching approach using a concatenated forward and reverse protein sequence database was employed for global FDR estimation and control. Searching parameters include: 1) Precursor ion mass tolerance: 20 ppm; 2) Product ion mass tolerance: 0.02 Da; 3) Maximal missed cleavages per peptide: 2; 4) Fixed modifications: carbamidomethylation of cysteine; 5) Dynamic modifications: Oxidation of methionine, Acetylation of peptide N-terminals. Peptide filtering, protein inference and grouping, and FDR control were accomplished by Scaffold v5.0.0 (Proteome Software, Inc.) The filtered peptide-spectrum match (PSM) list was exported. Protein quantification was performed using IonStar, an in-house developed MS1 ion current-based quantitative proteomics method [84]. Peptide quantitative features were first generated by a two-step procedure encompassing 1) Chromatographic alignment with ChromAlign for inter-run calibration of retention time (RT) shift; ii) Data-independent MS1 feature generation a direct ion-current extraction (DICE) method, which extracts ion chromatograms for all precursor ions with

corresponding MS2 scans in the aligned dataset with a defined m/z-RT window (10 ppm, 1 min). Both steps were accomplished in SIEVE v2.2 (ThermoFisher Scientific). Post-feature generation data processing was accomplished by UHR-IonStar v1.4 (https://github.com/JunQu-Lab/UHRIonStarApp) [85]. The filtered PSM list and the quantitative features database were first integrated by MS2 scan number to generate a list of annotated frames with peptide sequence assignment. The annotated frames were then subjected to dataset-wide normalization, principal component-based detection and removal of peptide outliers, and data aggregation to protein level. Protein quantification results were exported and manually curated and processed in Microsoft Excel.
